# Breast cancer cell migration is regulated through junctional adhesion molecule-A-mediated activation of Rap1 GTPase

**DOI:** 10.1186/bcr2853

**Published:** 2011-03-23

**Authors:** Elaine A McSherry, Kieran Brennan, Lance Hudson, Arnold DK Hill, Ann M Hopkins

**Affiliations:** 1 Department of Surgery, Royal College of Surgeons in Ireland, RCSI Education and Research Centre, Smurfit Building, Beaumont Hospital, Dublin 9, Ireland

## Abstract

**Introduction:**

The adhesion protein junctional adhesion molecule-A (JAM-A) regulates epithelial cell morphology and migration, and its over-expression has recently been linked with increased risk of metastasis in breast cancer patients. As cell migration is an early requirement for tumor metastasis, we sought to identify the JAM-A signalling events regulating migration in breast cancer cells.

**Methods:**

MCF7 breast cancer cells (which express high endogenous levels of JAM-A) and primary cultures from breast cancer patients were used for this study. JAM-A was knocked down in MCF7 cells using siRNA to determine the consequences for cell adhesion, cell migration and the protein expression of various integrin subunits. As we had previously demonstrated a link between the expression of JAM-A and β1-integrin, we examined activation of the β1-integrin regulator Rap1 GTPase in response to JAM-A knockdown or functional antagonism. To test whether JAM-A, Rap1 and β1-integrin lie in a linear pathway, we tested functional inhibitors of all three proteins separately or together in migration assays. Finally we performed immunoprecipitations in MCF7 cells and primary breast cells to determine the binding partners connecting JAM-A to Rap1 activation.

**Results:**

JAM-A knockdown in MCF7 breast cancer cells reduced adhesion to, and migration through, the β1-integrin substrate fibronectin. This was accompanied by reduced protein expression of β1-integrin and its binding partners αV- and α5-integrin. Rap1 activity was reduced in response to JAM-A knockdown or inhibition, and pharmacological inhibition of Rap1 reduced MCF7 cell migration. No additive anti-migratory effect was observed in response to simultaneous inhibition of JAM-A, Rap1 and β1-integrin, suggesting that they lie in a linear migratory pathway. Finally, in an attempt to elucidate the binding partners putatively linking JAM-A to Rap1 activation, we have demonstrated the formation of a complex between JAM-A, AF-6 and the Rap1 activator PDZ-GEF2 in MCF7 cells and in primary cultures from breast cancer patients.

**Conclusions:**

Our findings provide compelling evidence of a novel role for JAM-A in driving breast cancer cell migration via activation of Rap1 GTPase and β1-integrin. We speculate that JAM-A over-expression in some breast cancer patients may represent a novel therapeutic target to reduce the likelihood of metastasis.

## Introduction

Breast cancer accounts for approximately 30% of all female cancers diagnosed in the European Union and is the leading cause of female cancer deaths. Over 85,000 women (many in their reproductive and economically productive years) succumbed to the disease in 2006 [1]. Although there have been substantial improvements in breast cancer treatment, targeted adjuvant therapies are restricted to treating those patients whose tumor cells express high levels of the few targetable breast cancer molecular markers, namely the estrogen and HER2 (human epidermal growth factor receptor 2) receptors. It is therefore clear that further improvements are needed in the molecular understanding, diagnosis, and treatment of breast cancer.

Most breast cancers originate in the epithelial cells lining breast ducts. Epithelial cell polarity in normal ducts is maintained via intercellular multiprotein adhesion complexes, which facilitate adhesion and allow communication between neighboring cells. Loss of epithelial polarity and consequent disruptions in tissue architecture, a hallmark of de-differentiation, are early features of breast cancer and other malignancies [2]. Emerging evidence points toward an important role for proteins of the intercellular tight junction (TJ) complex in mediating tumorigenesis. To date, several TJ proteins have been shown to be dysregulated in breast carcinoma, with claudins-3 and -4 highly upregulated [3] and claudin-7 downregulated in *in situ *and invasive ductal carcinomas [4]. Furthermore, loss of the TJ-associated protein ZO-1 (zona occludens-1) in breast cancer correlates with both poor prognosis [5] and increased expression of proteinases important for tumor invasion [6]. Interestingly, adhesion/polarity proteins have recently been shown to be targeted by oncogenes (such as ERBB2 [7] and MYC [8]), resulting in the disruption of tissue organization often observed during cancer development. Together, these studies provide strong evidence that adhesion proteins may act as key regulators of breast cancer initiation and progression.

The junctional adhesion molecule (JAM) family of TJ proteins has important functions in numerous cellular adhesive processes, including intercellular junction assembly and cell polarity [9], cell morphology [10], platelet activation [11], and leukocyte migration [12]. Pathophysiologically, JAM-A has been linked to various inflammatory disorders [13-15] and, more recently, some cancers [16,17]. However, discordance exists regarding the specific role of JAM-A in breast cancer [18,19]. Our previous investigations into the role of JAM-A in breast cancer had analyzed levels of JAM-A expression in two cohorts of patients with invasive breast cancer. We demonstrated a novel and significant association between JAM-A overexpression in breast tissue and poor prognosis for patients with breast cancer [19]. Notably, patients whose tumors had high JAM-A expression levels were significantly more likely to develop metastasis. Given that migratory capacity is crucial for tumor cell dissemination and that JAM-A has established functions in leukocyte migration [12], we sought to determine the contribution of JAM-A to breast cancer cell migration *in vitro*. Our study has demonstrated that, likely owing to a concomitant reduction in expression of the migratory protein β1-integrin, antagonism or knockdown of JAM-A in MCF7 human breast cancer epithelial cells significantly decreases cell migration [20]. β1-integrin has been strongly linked with murine breast proliferation [21] and the formation of metastases in mouse models of breast cancer [22]. Accordingly, results from our [19] and other [23] tissue microarray studies have shown an association between high β1-integrin expression levels and poor prognosis in patients with breast cancer.

In this study, we further investigated the JAM-A signalling events that regulate β1-integrin-dependent migratory events in breast cancer cells. We demonstrated that reductions in cell migration following JAM-A functional antagonism or short interfering RNA (siRNA)-mediated gene knockdown are exerted via downstream effects on the activity of Rap GTPase1, a known activator of β1-integrins [24] and a regulator of breast tumorigenesis [25]. Furthermore, we present data suggesting that JAM-A may activate Rap1 indirectly through associations with downstream signalling proteins AF-6 and the guanine exchange factor, PDZ-GEF2. Finally, using *ex vivo *immunoprecipitation (IP) strategies in primary cultures generated from patients with breast cancer, we demonstrate that the JAM-A signalling complexes identified *in vitro *in MCF7 cells also exist in tissues of patients with breast cancer. Given both the regulatory influence of JAM-A on breast cancer cell migration *in vitro *and its link with metastasis in patient tissue samples [19], our studies suggest a role for JAM-A as a potentially important therapeutic target for the development of future breast cancer therapies.

## Materials and methods

### Cell culture

The MCF7 breast cancer cell line was obtained from the European Collection of Cell Cultures through Sigma-Aldrich (Poole, UK) and maintained in minimum essential medium (Sigma-Aldrich) supplemented with 10% fetal bovine serum (FBS) (Lonza, Basel, Switzerland), 2 mM L-glutamine, 50 U/mL penicillin, 50 μg/mL streptomycin (Invitrogen Corporation, Carlsbad, CA, USA), and 1% non-essential amino acids (Sigma-Aldrich). Cells were maintained at 37°C in humidified air with 5% CO_2_.

### Primary culture generation

Human breast tissue samples from lumpectomy or mastectomy patients were gathered with informed consent in protocols approved by the Beaumont Hospital medical ethics (research) committee and were used to generate mammary epithelial primary cell cultures with minor modifications from published methods [26]. In brief, tissue biopsies from within the tumor (T) and from histopathologically normal non-tumor margins (N) were incubated in penicillin/streptomycin/neomycin (Invitrogen Corporation); minced in Dulbecco's modified Eagle's medium/F12 (Sigma-Aldrich) containing penicillin/streptomycin/neomycin, 10% FBS, 10 μg/mL insulin, 5 μg/mL fungizone, 100 U/mL hyaluronidase 1-S, and 200 U/mL collagenase (Sigma-Aldrich); and agitated for 2 hours at 37°C. Cells were pelleted and washed before being cultured in mammary epithelial growth medium (Lonza) at 37°C with 5% CO_2_. Trypsin/ethylenediaminetetraacetic acid and soybean trypsin inhibitor were used to subculture confluent flasks. Cells were harvested after one passage to generate enough material for IP assays and one or two passages for protein isolation. Only patient-matched primary cultures were used (Table 1).

**Table 1:** Pathological parameters of human breast tissue primary cultures

Tissue	Age, years	Diagnosis	Grade	ER	PR	HER2
1T, 1N	53	IDC	2	+	+	-
2T, 2N	32	DCIS	NA	+	+	NA
3T, 3N	42	IDC	3	-	-	-

The estrogen receptor (ER), progesterone receptor (PR), and human epidermal growth factor receptor-2 (HER2) status of each patient's tissue culture was derived directly from the histopathology report on each case. DCIS, ductal carcinoma *in situ*; IDC, invasive ductal carcinoma; NA, not available.

### siRNA-mediated gene-expression knockdown

JAM-A siRNAs were transfected into MCF7 breast cancer cells using the N-TER nanoparticle siRNA transfection system (Sigma-Aldrich). In brief, cells (1.5 × 10^6 ^cells/10-cm dish) were seeded 16 hours prior to transfection. Cells were washed in phosphate-buffered saline (PBS) and transfected with nanoparticles containing 75 nM final concentration of JAM-A siRNAs, negative control siRNA, or mock (N-TER reagent only) in minimum essential medium. Assays were performed after transfection for 48 hours at 37°C with 5% CO_2_. Two alternative JAM-A siRNAs were used for all experiments. Representative results from a single siRNA are shown for clarity.

### Antibodies/inhibitors

All antibodies used for Western blot and immunofluorescence analyses were obtained from commercial sources: rabbit polyclonal anti-JAM-A (Zymed Laboratories Inc., now part of Invitrogen Corporation), MAb13 rat anti-β1 integrin (BD Biosciences, San Jose, CA, USA), rabbit anti-β2, rabbit anti-β3, rabbit anti-β4, rabbit anti-β5, rabbit anti-αV, rabbit anti-α4, rabbit anti-α5 integrins (Cell Signaling Technology, Danvers, MA, USA), rabbit anti-actin (Abcam, Cambridge, UK), rabbit anti-Rap1 (Millipore Corporation, Billerica, MA, USA), rabbit anti-AF-6 (Invitrogen Corporation), mouse anti-PDZGEF2 (Santa Cruz Biotechnology, Inc., Santa Cruz, CA, USA), and mouse J.10.4 inhibitory anti-JAM-A (Santa Cruz Biotechnology, Inc.). Horseradish peroxidase (HRP)-conjugated secondary antibodies were anti-mouse and anti-rat (Sigma-Aldrich) or anti-rabbit (Cell Signaling Technology). IgG isotype control antibodies were mouse, rabbit, and rat (Sigma-Aldrich). The Rap1 pharmacological inhibitor was GGTI-298 (Calbiochem/Merck, Darmstadt, Germany).

### Adhesion assay

Cell adhesion strips coated with fibronectin (which binds αVβ1 and α5β1 integrins) or bovine serum albumin (BSA)-control substrate (Millipore Corporation) were rehydrated in 96-well plates with PBS for 30 minutes at room temperature. Cells (1 × 10^5^/well) were added and incubated for 2 hours at 37°C and 5% CO_2_, washed in PBS, stained with 0.2% crystal violet (Sigma-Aldrich) in 10% ethanol for 5 minutes at room temperature, and rinsed in PBS. Cell-bound stain was solubilized by gently shaking with solubilization buffer (1:1, 0.1 M NaH_2_PO_4_:50% ethanol) for 5 minutes. Absorbance was measured at 560 nm on a microplate reader. Results from three independent experiments with three replicates per experiment were pooled.

### Migration assays

Transwell migration assays were conducted on MCF7 cells following transient JAM-A gene-expression knockdown. Transwell chambers (8 μM pore) were coated with 10 μg/mL fibronectin (Sigma-Aldrich) overnight at 4°C, washed in PBS, and rehydrated with serum-free media for 30 minutes at 37°C. Media were removed and cells (1 × 10^5^/chamber) were added to upper chambers with 15% FBS in lower chambers as a chemoattractant. Chambers were incubated for 3 hours at 37°C and 5% CO_2_. Migrated cells on the underside of the filter were fixed in 10% ethanol for 20 minutes prior to staining with 4,6-diamidino-2-phenylindole (DAPI) (Sigma-Aldrich) for 10 minutes at room temperature. Membranes were mounted on glass slides and cells were enumerated using Cell B software (Olympus, Tokyo, Japan) to analyze multiple fluorescent micrographs. Results from three independent experiments with three replicates per experiment were pooled.

Scratch-wound migration assays were conducted on wild-type MCF7 cells after treatment with inhibitors. Confluent cell monolayers in 24-well plates were preincubated for 2 hours with 5 μg/mL mouse anti-JAM-A J.10.4 antibody, 5 μg/mL MAb13 anti-β1 integrin antibody, 10 μM Rap1 inhibitor GGTI-298, appropriate isotype-control IgG, or dimethyl sulphoxide (Sigma-Aldrich) vehicle control. A scratch wound was made using a pipette tip. Media were replaced and wounds were photographed at 0, 2, and 6 hours. Scion Image software (Scion Corporation Ltd., Frederick, MD, USA) was used to measure closure of the wound over time by averaging six individual measurements of wound size for each wound at each timepoint. Results from three independent experiments with three replicates per experiment were pooled.

### Rap1 activity assay

Adherent cells were washed in Tris-buffered saline and then scraped and dounced in 1 mL of Rap1 activation lysis buffer containing 50 mM Tris-HCl, 0.5 M NaCl, 1% NP40, 2.5 mM MgCl_2_, and 15% glycerol (Millipore Corporation) and 1% protease and phosphatase inhibitors (Sigma-Aldrich). Lysates were incubated with 30 μg of Ral GDS-RBD (Rap-binding domain) agarose slurry (Millipore Corporation) for 45 minutes at 4°C. Beads were pelleted and washed in lysis buffer. Bound proteins were recovered by boiling at 95°C for 5 minutes in 40 μL of 2X sample buffer. Each immunoblot depicted is representative of three independent experiments, and densitometry was conducted on triplicate experiments. Densitometric data were used to determine the average active-to-total Rap1 protein expression over triplicate experiments.

### Western blotting

Cultured cells were washed in 10 mL of PBS, scraped, and dounced in Relax lysis buffer containing 100 mM KCl, 3 mM NaCl, 3.5 mM MgCl_2_, 10 mM HEPES pH 7.4, and 1% Triton-X100 as well as protease and phosphatase inhibitor cocktails (Sigma-Aldrich). Protein samples were separated by SDS-PAGE under reducing conditions by using Tris-glycine running buffer. After electrophoresis, proteins were transferred to nitrocellulose membranes (Optitran; Sigma-Aldrich). Membranes were blocked in 5% milk for 1 hour. Protein expression was detected by using primary antibodies incubated overnight at 4°C. Membranes were washed and incubated for 1 hour with HRP-conjugated secondary antibodies. Antigen-antibody complexes were detected by using Western Lightning Enhanced Chemiluminescence reagent (PerkinElmer, Waltham, MA, USA). Each immunoblot depicted is representative of three independent experiments, and densitometry was conducted on triplicate experiments.

### Immunoprecipitation

Protein extraction was conducted as above. Equal protein concentrations from control siRNA, JAM-A siRNA, and mock-transfected cells were subjected to JAM-A IP. Preclear was conducted via rotation of protein lysates for 1 hour at 4°C with protein G-sepharose beads (GE Healthcare, Chalfont St.Giles, Bucks, UK). IP antibodies (J.10.4 anti-JAM-A antibody or isotype control mouse IgG; 4 μg/mL) were rotated for 1 hour at 4°C. Bound antibody was retrieved by rotation with protein G-sepharose beads for 3 hours at 4°C. Beads were washed in Relax lysis buffer, and bound proteins were recovered by boiling at 95°C for 5 minutes in 40 μL of 2X sample buffer. Western blotting was performed as above for JAM-A, AF-6, PDZ-GEF2, Rap1, and β1-integrin. Each IP and associated immunoblot was conducted three times for cell lines and once each per primary culture.

### Statistical analysis

Averaged data from triplicate adhesion, transwell migration, and Western blot experiments were used to generate bar graphs depicting average values ± standard deviations, with paired Student *t *tests used to measure significance. For scratch-wound migration assays ± inhibitors, linear regression analysis was used to calculate any differences between treatments and controls.

## Results

### JAM-A knockdown decreases integrin-mediated cancer cell adhesion and migration

**Figure 1:**
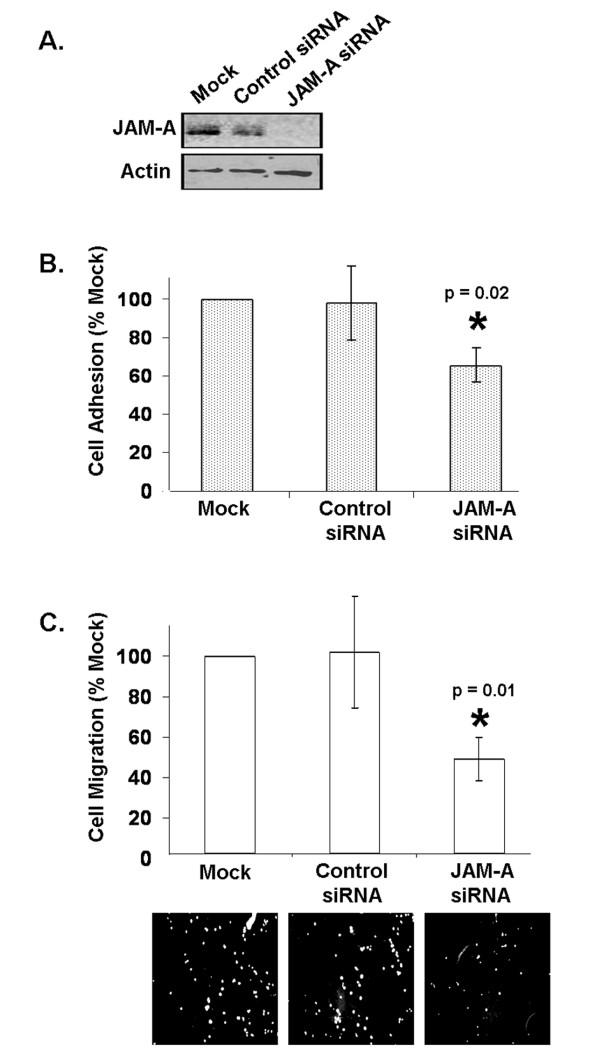
**JAM-A knockdown reduces breast cancer cell adhesion and migration**. **(a) **Transfection of MCF7 breast cancer cells with short interfering RNA (siRNA) targeting JAM-A reduces JAM-A protein expression. **(b) **Adhesion of MCF7 cells transfected with JAM-A siRNA to a fibronectin substrate was reduced relative to that induced under control conditions. **(c) **Individual cell migration of MCF7 cells transfected with JAM-A siRNA or control siRNA MCF7 cells across fibronectin-coated, 8-μm pore filters was reduced relative to control conditions. Images show DAPI (4'-6-diamidino-2-phenylindole)-stained migrated cells on transwell filters. Error bars refer to standard deviation of pooled triplicate experiments. JAM-A, junctional adhesion molecule-A.

Our previous study reported that high JAM-A expression levels in breast tissues from patients with invasive breast cancer correlated with reduced metastasis-free survival [19]. We and others have demonstrated that, likely owing to reductions in β1-integrin protein, knockdown of JAM-A gene expression in epithelial cells results in decreased collective cell migration [19,27,28] (Supplementary Figure S1A in Additional file 1). Indeed, integrins perform key roles at several key steps required for cell migration: adhesion assembly, disassembly, and turnover [29,30]. Taken together, our results to date suggest a role for JAM-A in promoting breast cancer cell migration through a β1-integrin-dependent pathway. To investigate this hypothesis, we used the MCF7 breast cancer cell line, which expresses high endogenous levels of JAM-A and β1-integrin. JAM-A protein levels were knocked down in these cells using a nanoparticle delivery system to transiently transfect cells with siRNA targeting JAM-A. Transiently-tranfected cells were analyzed after 48 hours for JAM-A knockdown at the protein level. JAM-A siRNA transfected cells displayed a 90% reduction in JAM-A protein expression (Figure 1a). Similar effects were observed with a second JAM-A siRNA construct (Supplementary Figure S1B in Additional file 1).

To investigate the link between JAM-A, β1-integrin, and cell migration, the ability of transfected cells to adhere to and migrate across a fibronectin substrate was analyzed (Figure 1b, c). Fibronectin binds multiple β1-integrin proteins through an RGD domain [31] and thus was used to facilitate analysis of the effects of JAM-A knockdown. First, transfected cells were allowed to adhere to fibronectin-coated transwell supports prior to cell staining and absorbance measurement. JAM-A knockdown cells showed an approximate 40% reduction in adhesion to fibronectin (Figure 1b) (*P *= 0.02) but not to control transwells coated with BSA (data not shown). Next, transfected cells were allowed to migrate across fibronectin-coated supports with 8-μm pores prior to fluorescent staining and enumeration. Here, in addition to showing a reduction in collective cell migration demonstrated in previous reports [19,27,28], JAM-A knockdown cells showed an approximate 50% reduction in individual cell motility in these matrix-specific transwell migration assays (Figure 1c) (*P *= 0.01), further underlining the important role of JAM-A in the regulation of breast cancer cell migration. However, JAM-A antagonism did not reduce invasion of MCF7 cells across Matrigel-coated filters in classic invasion assays (Supplementary Figure S2C in Additional file 2), owing to the fact that MCF7 cells are virtually non-invasive in this model (Supplementary Figure S2A in Additional file 2).

### JAM-A regulates β1-integrins and Rap1 GTPase in breast cancer cells

**Figure 2:**
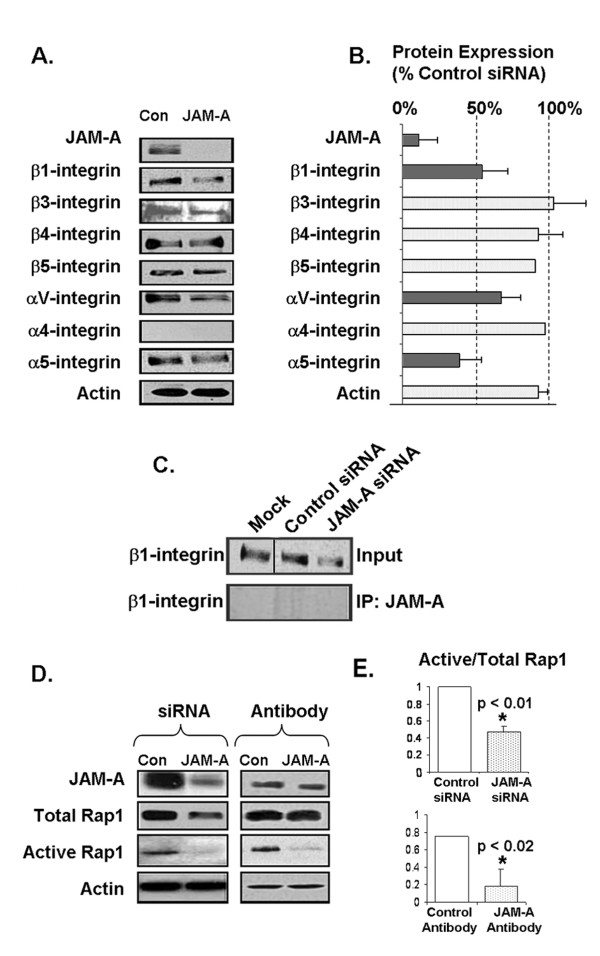
**JAM-A regulates β1-integrin protein expression and Rap1 GTPase activity in breast cancer cells**. **(a) **Western blot analysis of a panel of alpha- and beta-subunit integrins was conducted on MCF7 cell lines transfected with control short interfering RNA (siRNA) or JAM-A-siRNA. Assessment of actin expression was performed to control for protein loading. **(b) **Densitometry analysis of triplicate Western blot experiments showing relative protein expression of alpha- and beta-subunit integrins. Error bars refer to standard deviation of triplicate experiments. **(c) **β1-integrin Western blot analysis of equal total protein lysates (input) and JAM-A immunoprecipitates (IP:JAM-A) from MCF7 cells transfected with control siRNA or JAM-A-siRNA. **(d) **Western blot analysis of JAM-A, total Rap1, and active Rap1 protein expression in MCF7 cells transfected with control or JAM-A siRNA and in MCF7 cells treated with isotype control antibody or JAM-A inhibitory antibody. **(e) **Densitometric analysis of triplicate JAM experiments showing the ratio of active to total Rap1 after JAM-A knockdown or antagonism. Error bars refer to standard deviation of triplicate experiments. JAM-A, junctional adhesion molecule-A.

We and others have previously reported that JAM-A knockdown induces a concomitant reduction in β1-integrin in epithelial cells [10,19,27]. To probe whether this was a specific effect of JAM-A knockdown on β1-integrin in breast cancer cells, the expression of several integrin subunit proteins was analyzed following JAM-A knockdown in MCF7 breast cancer cells. Total protein isolates were prepared from MCF7 cells transfected with either JAM-A siRNA or control siRNA, and the protein expression levels of a panel of alpha- and beta-subunit integrins were assessed by Western blot analysis (Figure 2a, b). As previously stated, JAM-A siRNA transfectants achieved a greater than 90% reduction in JAM-A protein expression in comparison with control siRNA transfection. No change in the expression of β3-, β4-, or β5-integrins was observed upon JAM-A knockdown, as evidenced by densitometric analysis of triplicate immunoblots (Figure 2b). Expression of α4-integrin was not detected in MCF7 cells. Similar to previous studies [10,19,27], a 50% reduction in the expression of β1-integrin was observed upon transient JAM-A knockdown. Furthermore, reductions of approximately 30% and 60%, respectively, were observed for protein expression levels of αV- and α5-integrin subunits, both of which have been widely reported to complex with β1-integrin to form functioning fibronectin receptors [32]. These results further suggest that JAM-A exerts specific effects on β1-integrin heterodimers.

We next sought to investigate the signalling events connecting JAM-A engagement at the cell-cell interface to β1-integrin function at the cell-matrix interface. Given the spatial separation of these proteins, we hypothesized that their physical association was unlikely. To test this, JAM-A IPs were conducted on protein isolates from MCF7 cells transfected with JAM-A or control siRNA (Figure 2c). A reduction of β1-integrin total protein expression was confirmed in input samples of equal protein concentration. However, no bands were observed in JAM-A immunoprecipitated lanes immunoblotted for β1-integrin, confirming an absence of physical association between JAM-A and β1-integrin. We therefore concluded that other signalling proteins must be responsible for linking β1-integrin function to JAM-A.

One candidate protein, the integrin activator Rap1 GTPase [33], has been shown to regulate tissue polarity, lumen formation, and invasive potential in human breast epithelial cells [25]. We therefore investigated whether JAM-A knockdown using siRNA or functional inhibition using JAM-A inhibitory antibody altered Rap1 expression and activity (Figure 2d). Western blot analyses of MCF7 breast cancer cells showed a marginal decrease in total protein expression of Rap1 following JAM-A knockdown. However, a near abolition of active (GTP-bound) Rap1 was observed in JAM-A knockdown cells. In an effort to further confirm the regulation of Rap1 activation status by JAM-A, MCF7 cells were treated with an inhibitory antibody to JAM-A (J10.4). Western blot analysis demonstrated negligible changes in total Rap1 expression but dramatic reductions in active Rap1 following JAM-A inhibition. Indeed, reductions of 50% (*P *<0.01) and 80% (*P *<0.02) were observed for expression ratios of active Rap1 to total Rap1 following JAM-A knockdown and inhibition, respectively (Figure 2e), strongly indicating that JAM-A regulates downstream activation of Rap1. However, it is notable that the *ratios *of active-to-total Rap1 were not equivalent between knockdown and inhibition conditions, because JAM-A knockdown (but not inhibition) induced a reduction in the *total *levels of Rap1 protein.

### Inhibition of putative JAM-A signalling proteins reduces breast cancer cell migration

**Figure 3:**
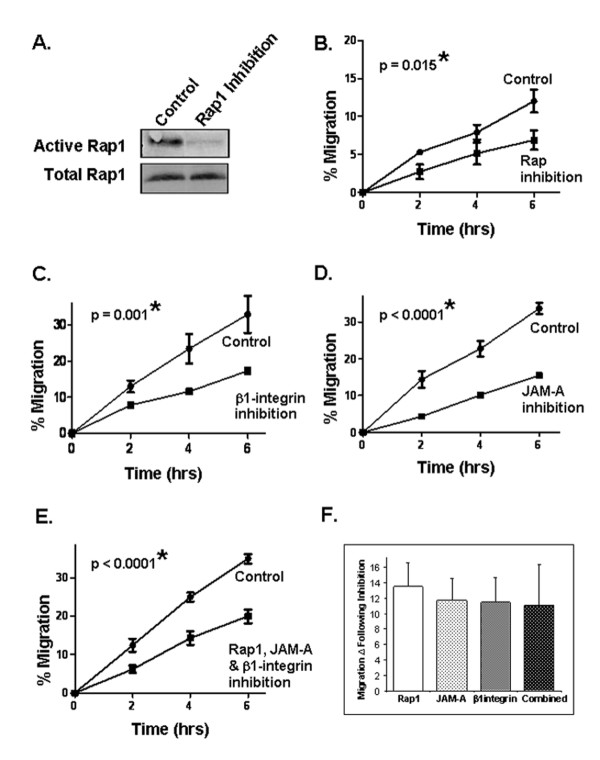
**Rap1 or β1-integrin protein antagonism reduces breast cancer cell migration**. **(a) **Western blot analysis of active Rap1 protein expression in MCF7 cells following treatment with the Rap1 pharmacological inhibitor GGTI-298 (10 μM). Migration of MCF7 cells in scratch-wound assays after pretreatment for 2 hours with Rap1 inhibitor **(b)**, MAb 13 anti-β1-integrin inhibitory antibody **(c)**, J104 anti-JAM-A inhibitory antibody **(d)**, or a combination of all three antagonists **(e)**. Error bars refer to standard deviation and represent triplicate values in a representative experiment. **(f) **Graphic representation of migration differences between control treatments and inhibitor/antibody treatments alone and in combination. Error bars refer to standard deviation in pooled triplicate experiments. JAM-A, junctional adhesion molecule-A.

Our results thus far suggested the existence of a JAM-A signalling pathway in MCF7 breast cancer cells, and that this pathway is initiated by JAM-A signalling via Rap1 and β1-integrin and culminates in cancer cell migration. We next reasoned that, if this pathway were linear, inhibition of any step should elicit inhibitory effects on cell migration similar to those induced upon inhibition of JAM-A alone. To investigate this hypothesis, we first treated MCF7 cells with the Rap1 pharmacological inhibitor GGTI-298 and verified that active Rap1 is reduced following treatment (Figure 3a). GGTI-298 also significantly reduced MCF7 cancer cell migration in scratch-wound assays (Figure 3b) (*P *= 0.015). Next, we demonstrated that treatment of MCF7 cells with an inhibitory antibody targeting β1-integrin elicited a similar reduction in MCF7 cell migration, from approximately 32% wound closure to 18% wound closure at 6 hours (Figure 3c) (*P *= 0.001). This was mirrored upon JAM-A inhibition, in which cell migration was observed to decrease from approximately 32% to 15% wound closure after 6 hours (Figure 3d) (*P *<0.0001). Combined treatment of MCF7 cells with inhibitors of Rap1, β1-integrin, and JAM-A resulted in a decrease in cancer cell migration from approximately 35% to 18% wound closure after 6 hours (Figure 3e) (*P *<0.0001). When the migratory differences between treatments and controls were quantitatively compared, no additive effects in response to inhibitor combinations versus single inhibitors alone were observed (Figure 3f). This indicated that JAM-A, Rap1, and β1-integrin are likely to function together in a linear signalling pathway in breast cancer cells.

### JAM-A co-associates with AF-6 and PDZ-GEF2

**Figure 4:**
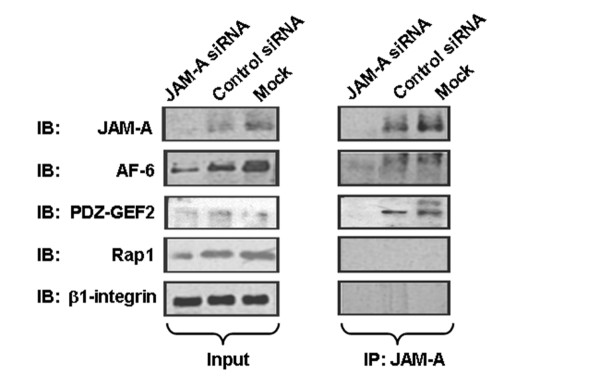
**JAM-A co-associates with AF-6 and PDZ-GEF2 in breast cancer cells**. Immunoblot (IB) analysis for JAM-A, Rap1, AF-6, and PDZ-GEF2 in equivalent concentrations of total protein lysates (input) and JAM-A immunoprecipitates (IP:JAM-A) from mock-transfected MCF7 cells and MCF7 cells transfected with control short interfering RNA (siRNA) or JAM-A-siRNA. JAM-A, junctional adhesion molecule-A.

Using IP strategies, we next sought to determine the JAM-A associations and signalling events that may affect downstream Rap1 activation. Total protein was extracted from MCF7 cells transfected with control siRNA or JAM-A siRNA, and JAM-A protein-binding partners were co-immunoprecipitated using an anti-JAM-A antibody (Figure 4). We first tested for the presence of Rap1 in JAM-A immunoprecipitates but did not detect any co-association. We therefore reasoned that intermediate signalling proteins must link JAM-A to the downstream activation of Rap1. Knockdown of the Rap1-associating protein [34] AF-6 (afadin) had been shown to result in reductions in active Rap1 and colorectal cancer cell migration [27]. We therefore investigated whether JAM-A co-associated with AF-6 in MC7F breast cancer cells. JAM-A immunoprecipitates from MCF7 protein lysates were immunoblotted with AF-6, and co-association between a pool of JAM-A and AF-6 was detected (Figure 4). Similar effects were observed using a second, independent JAM-A siRNA construct (Supplementary Figure S1B in Additional file 1). In addition to demonstrating a role for AF-6, previous studies had demonstrated a role for the Rap1 activator PDZ-GEF2 in both lung cancer cell adhesion [35] and colorectal carcinoma cell migration [27]. Similarly, JAM-A immunoprecipitates immunoblotted for PDZ-GEF2 confirmed co-association between a pool of JAM-A and PDZ-GEF2 in MCF7 breast cancer cells (Figure 4).

### JAM-A signalling complexes are altered in tissues of patients with breast cancer

**Figure 5:**
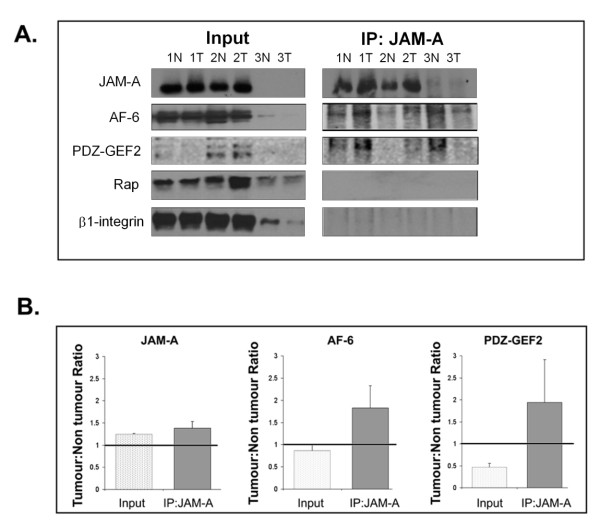
**JAM-A signalling is increased in breast cancer primary tumor cells**. **(a) **Immunoblot analysis for JAM-A, AF-6, PDZ-GEF2, Rap1, and β1-integrin protein expression conducted on input lysates of equivalent protein concentrations (left) and JAM-A immunoprecipitates (right) isolated from matched tumor (T) and non-tumor (N) primary breast cultures. **(b) **Comparison of tumor-to-non-tumor ratios from pooled samples of input protein and JAM-A immunoprecipitates for JAM-A, AF-6, and PDZ-GEF2 individually. JAM-A, junctional adhesion molecule-A.

Our accumulating results suggested a breast cancer signalling pathway in which JAM-A associates with AF-6 and PDZ-GEF2 to activate Rap1 and regulate β1-integrin-mediated cell migration. To determine the *ex vivo *relevance of this putative JAM-A signalling pathway, we sought to verify our results in primary cultures isolated from multiple tissues of patients with breast cancer (Table 1). Primary cultures were generated from three patient-matched tumor (1T, 2T, and 3T) and non-tumor (1N, 2N, and 3N) tissues, and total protein was extracted for investigation of JAM-A co-associations. JAM-A IP was conducted on all samples followed by immunoblotting for JAM-A, AF-6, PDZ-GEF2, total Rap1, and β1-integrin (Figure 5a). Analysis of total protein levels demonstrated a small increase in JAM-A expression in patient tumor cultures relative to non-tumor cultures (Figure 5a input and Supplementary Figure S3 in Additional file 3). Negligible differences in protein expression between tumor and non-tumor cultures were observed for AF-6, Rap1, and β1-integrin (Figure 5a input and Supplementary Figure S3 in Additional file 3). Notably, however, after densitometric pooling, tumor cultures displayed a reduced total protein expression of PDZ-GEF2 in comparison with non-tumor cultures (Supplementary Figure S3 in Additional file 3). Analysis of JAM-A immunoprecipitates demonstrated co-association between JAM-A and a pool of AF-6 and PDZ-GEF2 but not Rap1 or β1-integrin (Figure 5a), which mirrors data acquired from MCF7 breast cancer cells (Figure 4). Pooled densitometric analysis of tumor-to-non-tumor ratios following IP experiments revealed a trend toward increased association of both AF-6 and PDZ-GEF2 with JAM-A, suggesting enhanced formation of a JAM-A/AF-6/PDZ-GEF2 signalling complex in tumor cells (Supplementary Figure S3 in Additional file 3). Together, our findings provide compelling evidence of a novel role for the cell-cell adhesion protein, JAM-A, in influencing breast cancer cell migration at the cell-matrix interface via regulation of Rap1 and β1-integrin.

## Discussion

Although recent improvements in breast cancer treatments have resulted in average 10-year survival rates of approximately 72% [36], improved therapeutic strategies are required to target metastasis, the leading cause of death in patients with breast cancer. Loss of cell polarity is an early indicator of carcinoma progression [2]. To date, several proteins in cell polarity complexes [7,37] and cell junctional complexes [38,39] have been implicated in breast cancer [40]. Our previous studies demonstrated a novel and significant association between mammary overexpression of the TJ protein JAM-A and poor prognosis of patients with breast cancer [19]. This was initially surprising in light of another study reporting that *loss *of JAM-A promoted invasive behavior in breast cancer cell lines [18,19]. However, JAM-A likely plays a complex temporal role in cancer. Low JAM-A expression could potentially reduce adhesion and facilitate detachment of cells from early-stage tumors; later on, signalling events arising from high JAM-A expression may promote the migratory events associated with tumor invasion and metastasis. Accordingly, we demonstrated a link between JAM-A and β1-integrin protein expression and a regulatory influence of JAM-A on breast cancer cell migration *in vitro *[19]. The aim of the present study was to further elucidate the mechanisms whereby JAM-A influences cancer cell migration, in an attempt to explain the increased metastatic events observed in breast cancer patients whose tumors express high levels of JAM-A.

As presented in this article, our evidence is consistent with a pathway whereby JAM-A regulates β1-integrin-mediated breast cancer cell migration *in vitro *via alterations in key signalling proteins downstream of JAM-A. We have also verified that key JAM-related signalling complexes exist *ex vivo *in primary cultures isolated from tissues of patients with breast cancer, supporting the clinical relevance of our studies.

We first demonstrated that knockdown of JAM-A protein expression in MCF7 breast cancer cells significantly reduced breast cancer cell adhesion and migration. These results are not surprising, given the well-established functions of JAM-A in promoting epithelial cell spreading [10] and leukocyte migration [12]. However, it is important to note that JAM-A knockdown or antagonism did not *abolish *cell migration, indicating that this is not the sole regulatory pathway controlling a process as fundamental as cell migration.

As the integrin family of proteins are crucial regulators of both cell adhesion and cell migration [41], we probed a putative cross-regulation between JAM-A and integrins in breast cancer by measuring the expression levels of several alpha- and beta-subunit integrins in JAM-A knockdown MCF7 cells. Transient JAM-A knockdown specifically reduced the expression of β1-integrin and its alpha-subunit-binding partners αV and α5. Integrin knockdown was predictably not absolute, since integrins form the terminal step of migratory signalling cascades from multiple upstream regulators [42,43]. Nonetheless, our results suggested that JAM-A specifically affected β1-integrin heterodimers. Furthermore, previous studies had widely validated both αVβ1 and α5β1 integrins as functioning fibronectin receptors, thereby influencing our choices of substrate for migration and adhesion experiments [32]. Other evidence has shown that both αvβ1 and α5β1 integrins are RGD-binding integrins that recognize specific ligands containing an RGD tripeptide active site [32]. RGD-binding integrins bind to several ligands (both extracellular matrix and soluble), suggesting mechanisms to explain the influence of integrins on diverse cellular processes. Our results thus built upon studies in intestinal epithelial cells [10,27] to show that JAM-A may regulate β1-integrin-mediated migratory processes in breast cancer cells. This is consistent with our previous work suggesting a correlation between protein overexpression of JAM-A and β1-integrin in breast cancer both *in vivo *and *in vitro *[19]. Interestingly, the related family member JAM-C has also been shown to exert regulatory influence over β1-integrin activation and cell adhesion/migration via a motif in the JAM-C cytoplasmic tail [44].

β1-integrin has long been implicated in breast morphogenesis [45] and malignancy [46] via its regulatory influence on processes such as growth, apoptosis, migration, and invasion [20,47]. Indeed, inhibition of β1-integrin in three-dimensional breast cell cultures *in vitro *has been shown to phenotypically revert malignant cell aggregates to structures resembling normal breast acini *in vivo *[48]. Expression of β1-integrin in human breast cancer has been associated with poor patient survival [23] and resistance to both radiotherapy [49] and the adjuvant chemotherapy, trastuzumab (Herceptin) [50]. Given the pro-tumorigenic cellular processes governed by β1-integrin signalling and given the effect of JAM-A expression on β1-integrin protein expression, it was logical to hypothesize that JAM-A may directly regulate downstream β1-integrin-mediated processes.

To test this, we sought to determine whether JAM-A signalling culminates in β1-integrin activation in breast cancer cells. Our IP results showed no direct physical co-association between these two proteins. However, similar to studies in colonic epithelial cells [27], we found that JAM-A knockdown or inhibition in breast cancer cells significantly reduced the activity of Rap1 GTPase, a known activator of β1-integrins [24] and a regulator of cellular adhesion [51]. Some residual Rap1 activity was observed even in JAM-A knockdown or antagonized cells, consistent with the fact that there are multiple known upstream regulators of Rap1 function [51].

Rap1 GTPase protein activity is controlled by Rap guanine nucleotide exchange factors (GEFs) and Rap GTPase activating proteins (GAPs), which regulate guanine nucleotide exchange and the thus activity status of the protein [51]. Interestingly, increased Rap1 activity has been implicated in several cancer types, including thyroid [52] and prostate [53]. Downregulation of Rap1 GAP, a negative regulator of Rap1 activity, has been demonstrated in both pancreatic [54] and thyroid [52] cancer. Furthermore, activation of Rap1 in prostate cancer cells increases cell migration and invasion *in vitro*, and introduction of activated Rap1 in a xenograft prostate cancer mouse model has been reported to enhance metastasis [53]. To date, only one study (using mouse xenograft models and three-dimensional cell culture models) detailing the role of Rap1 in breast cancer has been published [25]. The study demonstrated that Rap1 is a regulator of breast architecture, that normal levels of activation maintain polarity during morphogenesis, and that increased activation induces tumor formation and breast cancer progression *in vivo*. Several studies have investigated Rap activity via Rap GAP loss or Rap GEF gain, but to our knowledge, none has focused on the role of possible upstream effector proteins such as JAM-A in breast cancer. However, it has been shown independently that both JAM-A [55] and Rap1 [56] proteins are required for fibroblast growth factor (FGF)-induced angiogenesis *in vitro*. Our results provide further evidence that Rap1 may represent an important downstream effector of JAM-A in the development and progression of breast cancer. That JAM-A, Rap1, and β1-integrin exist in a linear pathway was supported by evidence from migration assays in which combined pharmacological inhibition of all three proteins failed to exert additive effects relative to single inhibition of any one protein.

To further probe the association of JAM-A and Rap1 in breast cancer, IP experiments were conducted to identify direct binding partners that might physically link both proteins. Like the authors of a previous study [27], we were unable to detect direct co-association between JAM-A and Rap1. However, JAM-A co-associations were detected with both the adhesion protein AF-6 and the Rap GEF, PDZ-GEF2. AF-6 largely functions to connect membrane-associated proteins to the actin cytoskeleton [34] and is known to recruit and bind JAM-A at intercellular junctions via a PDZ domain [57]. AF-6 has also been suggested to be a Rap effector [58], and AF-6 knockdown in colonic epithelial cells reportedly decreases Rap1 activity and cell migration *in vitro *[27]. However, knockdown of AF-6 in T cells has been shown to enhance Rap1-induced integrin-mediated cell adhesion [34]. These contrasting roles for AF-6 are perhaps not surprising when one considers that maintenance of cell adhesion is crucial for epithelial cell polarity but that maintenance of a non-adherent state is favored for resting T-cell function. However, possible mechanisms accounting for this duality are still unknown. Little is known regarding the role of AF-6 in breast cancer, and only a single study, owing to an observed correlation between AF-6 loss and poor patient prognosis, suggests AF-6 as a potential tumor suppressor [59].

As mentioned above, GEF proteins such as PDZ-GEF2 are crucial activators of Rap1 GTPase [51]. Indeed, studies in colonic epithelial cells have demonstrated that knockdown of PDZ-GEF2 simultaneously reduces β1-integrin protein expression and cell migration [27]. Although there are no data regarding its expression or dysregulation in breast cancer, PDZ-GEF2 has been identified as an upstream activator of Rap1 required for the maturation of adherens junctions in lung carcinoma cells [35]. Given that dynamic adhesion changes are involved in migration and invasion, it is plausible that PDZ-GEF2 may also regulate these processes, which are critical for cancer progression.

In this study, we have presented results indicating a role for JAM-A in the regulation of β1-integrin-mediated migratory processes in breast cancer cells. Our data suggest a linear signalling pathway whereby JAM-A engagement leads to activation of Rap1 via PDZ-GEF2 and AF-6 and culminates in β1-integrin-induced cell migration. These results are similar to those from studies using colonic epithelial cells [27], indicating that this JAM-A signalling pathway may be conserved in several cell types. However, site-specific expression of JAM-A in mouse endothelial cells has been reported to prevent spontaneous motility *in vivo *[60], illustrating that the *in vivo *role of JAM-A in regulating migration is complex and spatially dependent. To exclude the possibility of an artefactual JAM-Rap-β1-integrin pathway in MCF7 cells, however, we have also verified our results in primary breast cell cultures isolated from tissues of patients with breast cancer. Our data revealed an increase in JAM-A protein expression in patient tumor cultures compared with non-tumor cultures, corroborating our previous study observing JAM-A overexpression in invasive breast cancer tissue microarrays [19]. Patient primary cultures also showed physical co-associations between JAM-A AF-6 and PDZ-GEF2 but not with Rap1 or β1-integrin. This mirrors *in vitro *cell line data from us and others [27] and further supports the possibility that JAM-A drives a pro-migratory pathway.

**Figure 6:**
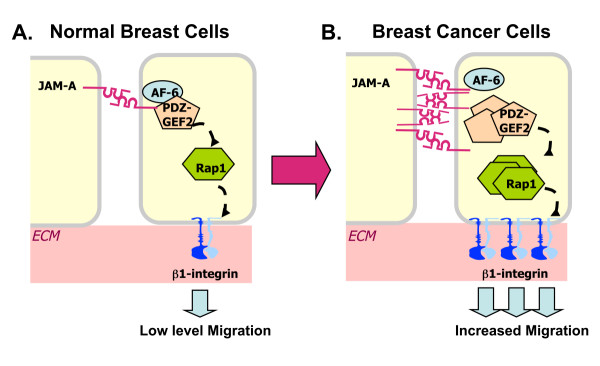
**Model of JAM-A signalling in human breast cancer cells**. Our working model hypothesizes that, in normal breast cells, a baseline level of JAM-A signalling via AF-6 and PDZ-GEF2 leads to a low level of β1-integrin-mediated cell migration required for crucial normal physiological processes such as wound healing **(a)**. However, in breast cancer cells, overexpression of JAM-A leads to its increased association with PDZ-GEF2 protein and this in turn hyperactivates the GTPase Rap1. We suggest that this culminates in increased β1-integrin-mediated cancer cell migration and leads to increased risk of invasion and metastasis **(b)**. ECM, extracellular matrix; JAM-A, junctional adhesion molecule-A.

Intriguingly, patient tumor primary cultures displayed a trend toward increased co-association of JAM-A with both AF-6 and PDZ-GEF2 in comparison with that in non-tumor cultures. This suggests the potential for increased signalling via an AF-6/PDZ-GEF2 pathway downstream of JAM-A overexpression in tumor cells. We speculate that this could lead to hyperactivation of Rap1 and consequent hyperactivation of β1-integrin (Figure 6). However, given the practical limitations of primary cultures (including a finite lifespan and slower growth and thus lower cell numbers in comparison with immortalized cultures), it was not feasible to conduct functional assays investigating integrin-mediated cell migration or invasion secondary to JAM-A protein manipulation. Future work (including animal models) will shed further light upon the mechanistic pathways involved in JAM-A signalling *in vivo*. Nonetheless, our current study has presented a model of JAM-A signalling in breast cancer cells and may help to explain the increase in metastatic events observed in breast cancer patients overexpressing JAM-A [19].

## Conclusions

Our findings provide compelling evidence of a novel role for the cell-cell adhesion protein JAM-A in influencing breast cancer cell migration. We have shown that JAM-A signals via Rap1 and β1-integrin proteins, both of which are crucial for cell adhesion, migration, and invasion. Furthermore, when taken in context with our previous study linking JAM-A and breast cancer, our *in vitro *data from cell lines and patient primary cultures suggest that JAM-A signalling may facilitate metastatic spread. In fact, a recent article has identified a micro-RNA (miR-145) whose overexpression reduces invasive and motile behavior in breast cancer cells by targeting JAM-A for downregulation [61]. We propose that JAM-A-mediated 'hijacking' of adhesive and migratory functions may represent a new therapeutic target for the development of anti-migratory cancer therapies.

## Abbreviations

BSA, bovine serum albumin; FBS, fetal bovine serum; GAP, GTPase activating protein; GEF, guanine nucleotide exchange factor; HRP, horseradish peroxidase; IP, immunoprecipitation; JAM, junctional adhesion molecule; PBS, phosphate-buffered saline; siRNA, short interfering RNA; TJ, tight junction.

## Competing interests

The authors declare that they have no competing interests.

## Authors' contributions

EAMcS participated in the design of the study, performed most of the experimental work, interpreted the data, and drafted the manuscript. LH performed the primary culture isolations. KB and ADKH participated in analysis and interpretation of the data. AMH conceived of the study, participated in its design, interpreted the data, and revised the manuscript. All authors read and approved the final manuscript.

## Supplementary Material

Additional file 1 **Supplementary Figure S1. Reduced cell migration downstream of JAM-A knockdown is reproducible with different siRNA constructs**. (A) Fold change in % wound closure over time of control MCF7 cells compared with cells transfected with two separate JAM-A siRNAs or a mock control siRNA. **(B) **Representative immunoblots illustrating expression levels of JAM-A, AF-6, and Rap1 (left panel) or co-precipitation of the same proteins with JAM-A (right panel) in MCF7 cells transfected with two separate JAM-A siRNAs or a mock control siRNA. Click here for file

Additional file 2 **Supplementary Figure S2. JAM-A inhibition reduces migration but not invasion of MCF7 cancer cells**. **(A) **Comparison of the relative invasion rates of MDA-MB-231, Hs579T cells and MCF7 cells across Matrigel-coated Transwell filters; confirming the non-invasive nature of MCF7 cells. Accordingly, although JAM-A antagonism with the inhibitory antibody J10.4 exerted a significant anti-migratory effect on MCF7 cells in scratch wound assays by 4 h **(B)**, no significant antagonism of MCF7 invasion across Matrigel-coated filters was observed even after 24 h exposure to J10.4 **(C)**. Click here for file

Additional file 3 **Supplementary Figure S3. Pooled analysis of JAM-A signalling proteins in tumour versus normal breast tissue primary cultures**. **(A) **Ratio of pooled tumor to non-tumor densitometric values from JAM-A, AF-6, PDZ-GEF2, Rap1 and β1-integrin protein immunoblots with equal total input protein concentrations. **(B) **Ratio of pooled tumor to non-tumor densitometric values from JAM-A, AF-6, PDZ-GEF2, Rap1 and β1-integrin protein immunoblots of JAM-A immmunoprecipitates. Click here for file
