# Supplementary figures and images for: Breast cancer cell migration is regulated through junctional adhesion molecule-A-mediated activation of Rap1 GTPase
Source: Breast Cancer Res. 2011 Mar 23;13(2):R31. doi: 10.1186/bcr2853 (PMC3219194; doi:10.1186/bcr2853)

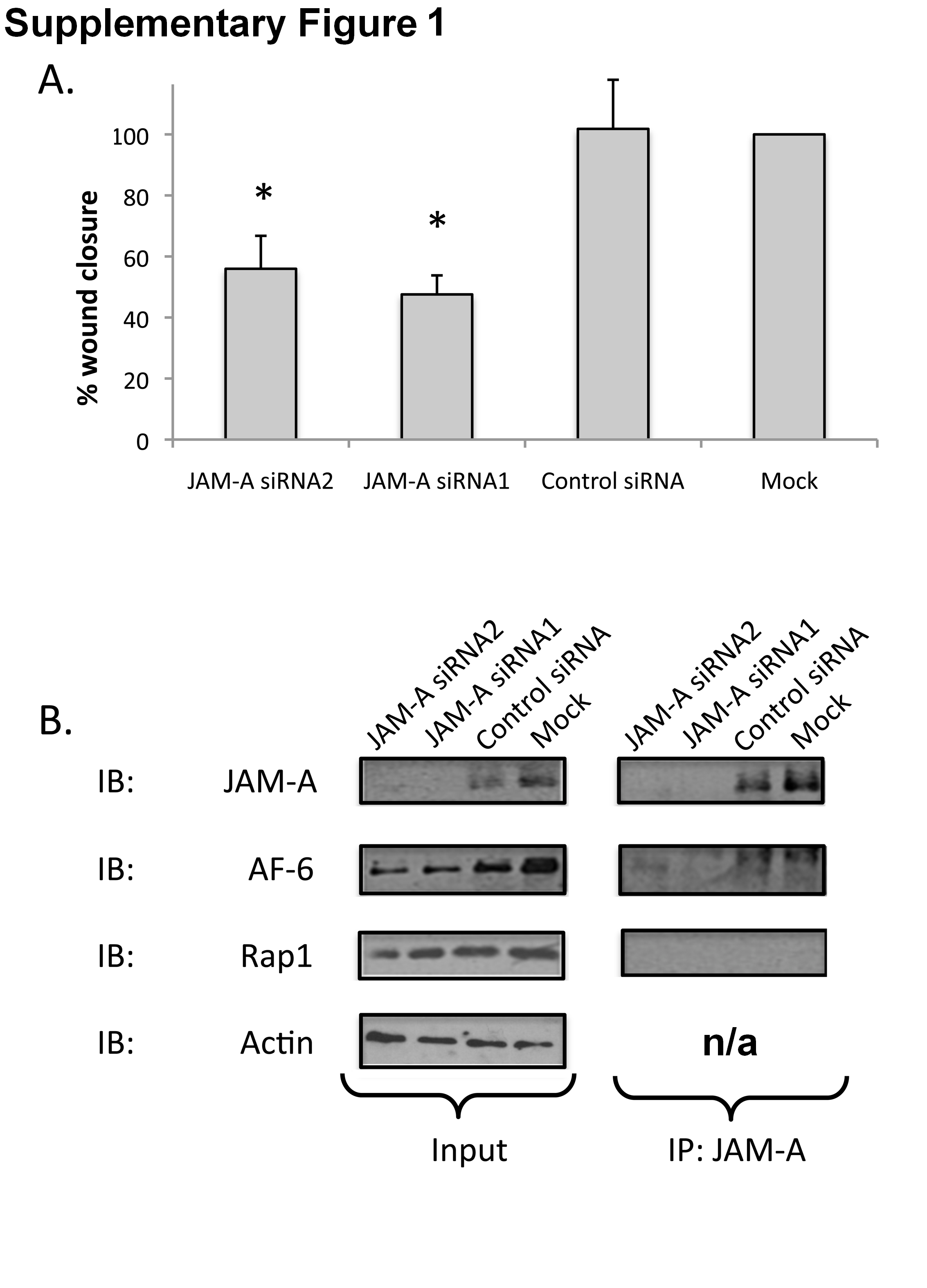

Supplement: Additional file 1 — Supplementary Figure S1. Reduced cell migration downstream of JAM-A knockdown is reproducible with different siRNA constructs. (A) Fold change in % wound closure over time of control MCF7 cells compared with cells transfected with two separate JAM-A siRNAs or a mock control siRNA. (B) Representative immunoblots illustrating expression levels of JAM-A, AF-6, and Rap1 (left panel) or co-precipitation of the same proteins with JAM-A (right panel) in MCF7 cells transfected with two separate JAM-A siRNAs or a mock control siRNA. [file bcr2853-S1.TIFF]

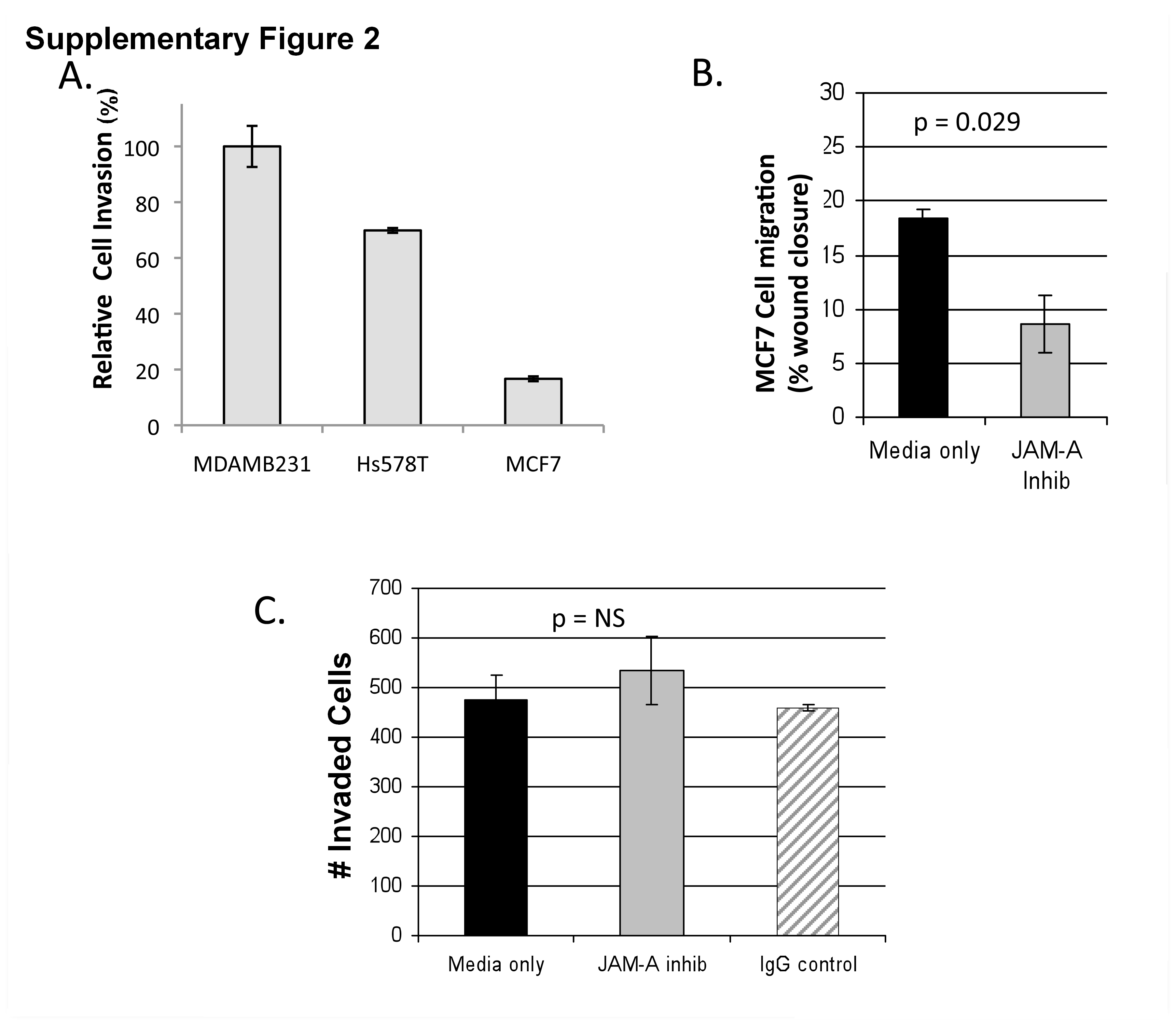

Supplement: Additional file 2 — Supplementary Figure S2. JAM-A inhibition reduces migration but not invasion of MCF7 cancer cells. (A) Comparison of the relative invasion rates of MDA-MB-231, Hs579T cells and MCF7 cells across Matrigel-coated Transwell filters; confirming the non-invasive nature of MCF7 cells. Accordingly, although JAM-A antagonism with the inhibitory antibody J10.4 exerted a significant anti-migratory effect on MCF7 cells in scratch wound assays by 4 h (B), no significant antagonism of MCF7 invasion across Matrigel-coated filters was observed even after 24 h exposure to J10.4 (C). [file bcr2853-S2.TIFF]

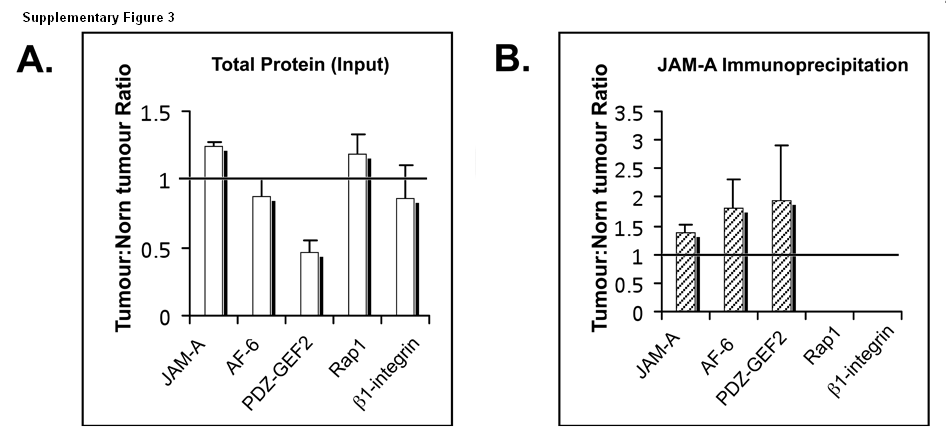

Supplement: Additional file 3 — Supplementary Figure S3. Pooled analysis of JAM-A signalling proteins in tumour versus normal breast tissue primary cultures. (A) Ratio of pooled tumor to non-tumor densitometric values from JAM-A, AF-6, PDZ-GEF2, Rap1 and β1-integrin protein immunoblots with equal total input protein concentrations. (B) Ratio of pooled tumor to non-tumor densitometric values from JAM-A, AF-6, PDZ-GEF2, Rap1 and β1-integrin protein immunoblots of JAM-A immmunoprecipitates. [file bcr2853-S3.TIFF]
